# Pharmacological Risk Factors for Delirium after Cardiac Surgery: A Review

**DOI:** 10.2174/157015912803217332

**Published:** 2012-09

**Authors:** Lurdes Tse, Stephan KW Schwarz, John B Bowering, Randell L Moore, Kyle D Burns, Carole M Richford, Jill A Osborn, Alasdair M Barr

**Affiliations:** a Department of Anesthesiology, Pharmacology & Therapeutics, The University of British Columbia, 2176 Health Sciences Mall, Vancouver, B.C., Canada, V6T 1Z3; b Department of Psychiatry, The University of British Columbia, Canada

**Keywords:** Cardiac surgery, delirium, drugs, medications, prevent, risk factors.

## Abstract

**Purpose:**

The objective of this review is to evaluate the literature on medications associated with delirium after cardiac surgery and potential prophylactic agents for preventing it.

**Source:**

Articles were searched in MEDLINE, *Cumulative Index to Nursing and Allied Health*, and EMBASE with the MeSH headings: delirium, cardiac surgical procedures, and risk factors, and the keywords: delirium, cardiac surgery, risk factors, and drugs. Principle inclusion criteria include having patient samples receiving cardiac procedures on cardiopulmonary bypass, and using DSM-IV-TR criteria or a standardized tool for the diagnosis of delirium.

**Principal Findings:**

Fifteen studies were reviewed. Two single drugs (intraoperative fentanyl and ketamine), and two classes of drugs (preoperative antipsychotics and postoperative inotropes) were identified in the literature as being independently associated with delirium after cardiac surgery. Another seven classes of drugs (preoperative antihypertensives, anticholinergics, antidepressants, benzodiazepines, opioids, and statins, and postoperative opioids) and three single drugs (intraoperative diazepam, and postoperative dexmedetomidine and rivastigmine) have mixed findings. One drug (risperidone) has been shown to prevent delirium when taken immediately upon awakening from cardiac surgery. None of these findings was replicated in the studies reviewed.

**Conclusion:**

These studies have shown that drugs taken perioperatively by cardiac surgery patients need to be considered in delirium risk management strategies. While medications with direct neurological actions are clearly important, this review has shown that specific cardiovascular drugs may also require attention. Future studies that are methodologically consistent are required to further validate these findings and improve their utility.

## INTRODUCTION

Delirium after cardiac surgery is associated with serious long-term medical consequences [1, 2] and high costs [3]. It affects approximately 30% of cardiac surgery patients, although reported incidences are variable [4-11]. Delirium is an acute and fluctuating state of confusion and disorientation, characterized by changes in attention, cognition, consciousness, and perception, and is often associated with changes in sleep patterns. Diagnosis is typically based on clinical assessments of patient symptoms, which are defined by the *Diagnostic and Statistical Manual* fourth, text-revised (DSM-IV TR) edition [12]. 

Factors that have previously been identified as key, independent predictors of delirium after cardiac surgery include advanced age, pre-operative cognitive decline, atrial fibrillation, previous delirium as well as a sizeable list of other conditions and co-morbidities [13-15]. Given that many of these risk factors are non-modifiable, emphasis recently has been on identifying risk factors that can be modified so that the incidence or severity of postoperative delirium may be reduced. A focus of such modifiable risk factors includes medications that cardiac surgery patients consume before, during, and after surgery. Interestingly, it seems that while some medications may be associated with a higher rate of delirium, others may be associated with a significantly reduced rate of delirium. Despite a growing number of studies that include perioperative drug use in their investigations, the influence of drugs on the development of delirium after cardiac surgery has not been the subject of critical review. 

Critical examination of perioperative drugs is important because these agents may have pharmacological actions (particularly within neural tissues) that can greatly influence the etiology of postoperative delirium. One popular theory for delirium etiology is the neurotransmitter hypothesis. This theory postulates that decreased neuronal metabolism from oxygen deprivation during cardiac surgery alters neuro-transmitter function and causes generalized dysfunction in the brain [16, 17]. In particular, the neurologically ubiquitous cholinergic system is believed to be deficient in the delirious patient [18, 19]; additionally, there may also be excesses of dopamine, norepinephrine, and glutamate, while serotonin and GABA levels may be increased or decreased [16, 19]. Therefore, cardiac medications such as digoxin, furosemide or nifedipine (which have relatively significant anticholinergic properties), and other medications like selective serotonin reuptake inhibitors (SSRIs), antipsychotics, or benzodiazepines, may pay important contributions to delirium etiology through these neurotransmitter pathways.

It is important to emphasize that most studies that have collected data on perioperative use of medications in delirious patients are prospective or retrospective observational studies, and therefore cannot imply direct causal relationships between the medications that were studied and the outcome of delirium. Even though the majority of these observational studies use multivariate logistic regression analyses to identify drugs that may independently increase or decrease the risk of delirium, such techniques cannot account for all the reasons why groups differ in the rates of delirium; depending on the covariates that are being controlled for in the regression models, there may be significant cross-study differences in the results. 

Another challenge of using the observational study design for delirium research is in determining the influence of intraoperative drugs on delirium. The reason for this is because behavioural abnormalities that appear immediately following surgery may be attributed to the residual effects of anesthesia, and patients may experience a form of delirium known as *emergence delirium*, which is a state of short-lived, self-limiting agitation that is attributable to substance use [20]. In the literature, emergence delirium is not consistently defined, and its etiological and pathological distinction from postoperative delirium is not consistently differentiated (compare, for example, [20] to [21]). For this reason, a couple of the studies that were reviewed for this synopsis did not commence their assessments of delirium in patients until the second day after surgery [5, 7]. Nevertheless it has been suggested that intraoperative factors reliably contribute to the development of postoperative delirium because the first symptoms almost always occur in the period shortly after awakening from sedation [13].

Similarly, it is also difficult to establish the role of postoperative drugs on delirium with such observational designs. Even though delirium is defined by the DSM-IV-TR as having multifactorial etiology (which includes preoperative, intraoperative, and postoperative factors) the role of postoperative factors on delirium is frequently downplayed in studies because they are not commonly considered ‘predictors’ of delirium, despite the fact that they are potentially modifiable. For instance, Afonso *et al*. [10], Katznelson *et al*. [22] and Redelmeier *et al*. [24] did not include any postoperative variables in their investigations, while Tan *et al*. [25], Shehabi *et al*. [26], and Tully *et al*. [27] focused primarily on pre- and intraoperative factors in their studies. Thus, studies that look at postoperative medications as potential factors that influence delirium are limited in number. 

Other studies that have looked at the influence of drugs on delirium are randomized controlled trials (RCTs). These studies, which are more robust, are typically designed to investigate the prophylactic effectiveness of certain medications like dexmedetomidine or rivastigmine to prevent delirium in vulnerable individuals. Unlike observational studies, well-controlled RCTs can suggest that any observed differences in the rates of delirium are due to differences in drug administration.

In this review, delirium after cardiac surgery is considered distinct from other types of postoperative deliria for a number of reasons. For one, different surgical populations often have different medication profiles and require different anesthesia techniques. Thus, the pharmacological triggers of delirium will vary depending on surgery. Secondly, the use of cardiopulmonary bypass (CPB) in cardiac surgeries requires special consideration since its use is associated with postoperative effects on neurological function and an increase in delirium [13]. Lastly, it is unknown if the pathophysiology of different postoperative deliria differs: research has shown that predictors of delirium appear to vary depending on surgery type, and levels of certain biomarkers for delirium also appear to vary with different forms [23]. 

The purpose of this article hence is to synthesize the evidence in the literature for drugs that have been shown to be associated with either a higher or a lower rate of delirium after cardiac surgery. We also discuss studies that have attempted to use certain drugs for strategic prevention of postoperative delirium.

## METHODS

Studies were searched in MEDLINE (January 1948 through January 2011), Cumulative Index to Nursing and Allied Health (CINAHL; 1982-2011), and EMBASE (1980-2011). MeSH headings that were searched included the following: delirium, cardiac surgical procedures, and risk factors. Keyword searches included: delirium, cardiac surgery, risk factors, and drugs. Reference lists of the articles that were retrieved were also searched for relevant citations. Articles were included only if they were published between January 2000 and December 2010, used DSM-IV-TR criteria or a standardized assessment tool (e.g., CAM, CAM-ICU, MDAS, ICDSC) for the screening or diagnosis of delirium, involved more than one assessment of delirium in the postoperative period, included only adults, were written in English, were prospective, retrospective, or interventional in their designs, and included cardiac procedures requiring CPB, i.e., coronary artery bypass grafting (CABG), valve replacements, combination CABG-valve replacements, or heart transplants^1^. The DSM-IV-TR, which is the current edition of the manual as of this publication, was published in 2000; therefore, only studies that were published in 2000 onward were included in this review. Studies were excluded if they took place in a community setting, if delirium was not a specific outcome, if the diagnosis of delirium was based solely on a clinical diagnosis without the use of DSM criteria or a standardized tool, or if they were reviews or case reports. 

It should be mentioned that the study by Redelmeier *et al*. [24] possesses some qualities that should exclude it for review; only 3.3% of the patient sample actually received cardiac interventions on CPB, with the rest receiving non-cardiac surgeries. It also used a retrospective diagnosis of delirium based on International Classification of Diseases (ICD) criteria instead of DSM-IV-TR criteria or a standardized assessment tool. However, Redelmeier *et al.* [24] did include an impressively large number of patients in their cohort (total *n* = 284, 158; cardiac patients *n* = 9,272), and this gives the study considerable relevance for cardiac surgery despite the small proportion of the total sample size that were actually cardiac surgery patients. More importantly, when the rate of delirium was analyzed with respect to their primary factor of interest (i.e., preoperative statin use), they found that the type of surgery received did not affect the relationship that they found between preoperative statin use and postoperative delirium [24] (this finding is further discussed below). For these reasons, and given the importance of the findings from the Redelmeier *et al. *[24] study, it was included for review. 

Most studies reviewed were not specifically designed to measure the role of drugs on postoperative delirium; in fact, this was only the case for 5 of the studies reviewed [3, 8, 9, 22, 26]. The majority of studies that collected data on medication administration included only a small number of specific drugs or general drug classes as variables in their extensive databases. These drug variables were developed *a priori*, or were included through naturalistic intake**(the only exception to this is the report by Burkhart *et al*. [11], which was a post-hoc analysis of the results from a randomized controlled trial performed by Gamberini *et al*., [8]).**For the most part, drug variables in these studies were quantified by comparing the proportions of delirious versus non-delirious individuals who were taking these drugs. The studies by Burkhart *et al.*, [11] and Hudetz *et al*. [9] also quantified and compared the doses of certain drugs consumed by delirious versus non-delirious individuals. 

From the studies that were evaluated, several drugs that patients were taking as outpatients prior to surgery, during surgery, and in postoperative intensive care emerged as being either significantly predictive or protective of postoperative delirium. In most studies, drugs that were identified to be significantly associated with delirium by univariate analysis were further analyzed by multivariate logistic regression to identify drugs that were independently associated with delirium. One study that took a rather different approach was by Katznelson *et al*. [22], who performed stepwise model selection procedures in which statins, their primary factor of interest, was always forced into the models regardless of its association (or lack thereof) with delirium in univariate analysis. Some drugs failed to demonstrate any significant associations with postoperative delirium in either direction in these studies, and these are also discussed.

Eight prospective observational studies, 5 randomized controlled trials, 1 retrospective observational study and 1 post-hoc analysis were selected for review, and these are summarized in Table **1**.

## RESULTS

See Table **2** for a complete summary of the drugs that have been studied in relation to delirium after cardiac surgery.

### I Preoperative Period

#### Psychiatric Drugs

Preoperative use of psychiatric medications is frequently cited to be a risk factor for postoperative delirium [28]. However, in the studies that met inclusion criteria for this review, only antipsychotics, antidepressants and benzodiazepines have been studied for their relationship to delirium [10, 24, 27]. 

Only one single study included preoperative antipsychotic use as a factor in their analysis. Redelmeier *et al*. [24] found that patients who took preoperative antipsychotics within the year before surgery were 1.57 times more likely to develop delirium after surgery than patients who were not taking antipsychotics (OR = 1.57, 95% CI 1.26 – 1.95; *p* < 0.001). This result was obtained by controlling for age, sex, social status, prior admissions, prior use of medications, all neuropsychiatric, cardiac, vascular and miscellaneous medications, and duration and type of surgery in their multivariable logistic regression model [24]. No other study that was evaluated for this review included preoperative antipsychotics in their investigations.

The work on antidepressants and delirium is more extensive, but likewise unconvincing due to small sample sizes and conflicting findings. Tully *et al*. [27] looked at preoperative tricyclic antidepressant use in a cohort of prospectively recruited cardiac surgery patients, but found no statistically significant effect of this class of drugs on the outcome of delirium. However, when individuals taking SSRIs were analyzed, use of these drugs was significantly associated with an increased rate of postoperative delirium compared to individuals who were not taking SSRIs (*p* = 0.01) [27]. Despite this association, the study faced statistical issues, as there was only one non-delirious individual in this study who was taking SSRIs. Hence, use of SSRIs was not included as a factor in multivariable logistic regression analyses in this study. To mitigate this issue, the authors formed a composite variable that included SSRIs, tricyclics, and drugs with known anticholinergic properties [27]. This composite variable not only significantly correlated with delirium (*p* ≤ 0.01), but also was reported to be highly predictive: patients who were taking SSRIs or tricyclic antidepressants and/or drugs with known anticholinergic effects were 5.12 times more likely to become delirious than patients who were not taking these drugs (95% CI 1.46-17.94; *p *= 0.01) [27]. A study by Afonso *et al*. [10] also included preoperative SSRI use in their prospective examination of potential predictors of postoperative delirium in cardiac surgery patients. However, because SSRIs were not frequently used in this patient sample (only one patient for in this sample was taking an SSRI), this drug was not included in univariate analysis and no conclusions could be drawn from these results. The study by Redelmeier *et al*. [24] also investigated the relationship between preoperative antidepressant use and postoperative delirium. Unlike the previous two studies, the retrospective design of the Redelmeier *et al. *[24] study facilitated the inclusion of a much larger sample size (*n* = 287, 353), and they were able to acquire a much larger number of patients who were taking antidepressants in the preoperative period (*n* = 266, 519). Redelmeier *et al*. [24] found that taking preoperative antidepressants in the year leading up to surgery doubled the odds of developing postoperative delirium in their patient sample (OR = 2.01, 95% CI 1.75 – 2.25; *p* < 0.001). 

Afonso *et al*. [10] included benzodiazepine use in their study but similar to their finding on SSRIs, they were unable to draw any conclusions since only one individual in this sample was taking a benzodiazepine in the preoperative period. Conversely, Redelmeier *et al*.’s [24] multivariable logistic regression model showed that preoperative use of benzodiazepines within the year before surgery was independently associated with a 1.40 times increase in the rate of delirium (OR = 1.40, 95% CI 1.28 – 1.53; *p* < 0.001). Thus, the findings for the influence of preoperative benzodiazepine use on postoperative delirium are inconclusive and further work is required to reveal any relationship that may exist.

No studies were found that included preoperative use of mood stabilizers, stimulants, anxiolytics or any other psychiatric drugs. 

#### Opioids

Preoperative opioids have not been thoroughly investigated for their relationship to delirium. Only one study meeting inclusion criteria included this as a factor [29]. Koster *et al*. [29] showed that preoperative use of opioids was not linked to postoperative delirium after cardiac surgery in their study (*p* = 1.00). However, given that only four individuals in the entire cohort of 112 patients were taking opioids before surgery [29], this finding bears the same limitations of sample size as the studies by Afonso *et al*. [10] and Tully *et al*. [27]. 

#### Anticholinergics

Two studies evaluated the relationship between preoperative anticholinergic drug use and postoperative delirium, and neither found an association between anti-cholinergics and delirium. These studies by Tan *et al*. [25] and Tully *et al*. [27] were both prospective, observational studies with patient samples of similar mean ages that were undergoing CABG, valve, or combined CABG-valve surgeries on CPB. Medication consumption data from both studies were recorded in terms of frequencies of drug use. Tan *et al*. [25] used the variable “other anticholinergic meds”, while Tully *et al*. [27] included “anticholinergic” drugs as a factor, but neither study precisely defined these variables, nor did they report the specific drugs that were included in these broad classes. As there is a wide range of drugs with direct or indirect anticholinergic activity [30-31], it would have been important for these studies to report exactly which drugs were included under these broad variables. Given this, neither Tan *et al*. [25] nor Tully *et al*. [27] found a significant relationship between drugs with anticholinergic activity and delirium after cardiac surgery (*p* = 0.10; *p* = 0.31 respectively). Though Tan *et al*. [25] did have a moderate number of patients who were taking preoperative anticholinergic medications in their study (*n* = 20 out of a total *n* = 53), the broad definition that they attributed to the variable “ other anticholinergic meds” means that additional studies are required for evidence on the effects of anticholinergic medications on postoperative delirium.

Interestingly, as noted above, when Tully *et al*. [27] grouped anticholinergic agents, SSRIs, and tricyclic antidepressants into a single composite variable, they found that patients who were taking any of these drugs preoperatively were approximately 5 times more likely to develop delirium after cardiac surgery than patients who were not taking these drugs. Considering that SSRIs and tricyclic antidepressants such as paroxetine and nortriptyline have been demonstrated *in vitro* to have moderate anticholinergic activity at therapeutic doses [31], it may be justified to regard this composite variable as a crude measure of the relationship between anticholinergic activity and the occurrence of postoperative delirium. Future studies would benefit from standardized quantification of anticholinergic medication burden (such as the anticholinergic drug scale [32]) to allow for comparisons to be made between delirious and non-delirious patients and to establish consistency across studies.

#### Statins

Studies that have looked at the influence of preoperative statin use on the outcome of delirium after cardiac surgery have produced contrasting findings: of the four studies that focused specifically on preoperative statin use, one study showed that it was protective against delirium [22], another showed that it was predictive of delirium [24], and two did not find a statistically significant relationship between lipid-lowering agents and delirium [9, 11]. Non-statin lipid lowering agents such as fenofibrate were investigated in only one study [24], and no relationship was found between these drugs and delirium. In evaluating these studies on statins, it is important to consider the major differences in methodologies and patient samples that make comparing results between these studies difficult. The study by Katznelson *et al*. [22] was a prospective observational study that was performed on patients undergoing cardiac surgery with CPB, while Redelmeier *et al*. [24] performed a retrospective analysis on patients undergoing either cardiac or non-cardiac elective surgeries, and used ICD criteria instead of DSM-IV-TR criteria for the diagnosis of delirium.

Redelmeier *et al*. [24] found that postoperative delirium was more likely to occur amongst patients taking preoperative statins than amongst those who were not taking statins (OR = 1.30, 95% CI 1.15 – 1.47; *p* < 0.001). To control for the broad range of surgeries that were included in this study, surgery type was included as a covariate in the analysis. This produced an OR = 1.12, with a 95% confidence interval of 0.99 – 1.27 (*p* = 0.07), meaning that surgery type had no effect on the relationship between statin use and delirium [24]. Redelmeier *et al*. [24] then proceeded to look at the relative risk of delirium exclusively amongst statin users. In this case, surgery type did significantly affect delirium: specifically, statin use was only independently associated with delirium for patients receiving non-cardiac surgeries (RR = 1.33, 95% CI 1.16 – 1.53), but not for patients receiving cardiac surgeries (RR = 1.26, 95% CI 0.86 – 1.87) [24]. It is pertinent to acknowledge that these results were only obtained when the factors of age, sex, duration of surgery, and “individual medications” (which was not explicitly defined in this report) were selected as covariates [24]. Had they picked other appropriate covariates, it is likely that they would have found different results. Redelmeier *et al*. [24] also found that the risk of delirium was increased for both simvastatin and atorvastatin (OR 1.46, 95% CI 1.15 – 1.84 for simvastatin; OR 1.68, 95% CI 1.34 – 2.09 for atorvastatin), but no association was observed for the non-lipophilic pravastatin (OR 1.26, 95% CI 0.96 – 1.64). No other cardiac medications were found to increase the risk of developing delirium after cardiac surgery in this study [24]. 

On the other hand, the prospective study by Katznelson *et al*. [22] came to the opposite conclusion. Cardiac surgery patients who were taking preoperative statins (mostly atorvastatin in this sample) had half the risk of developing delirium when compared to individuals who were not taking statins (OR = 0.54, 95% CI 0.35-0.84, *p* < 0.01) [22]. This relationship, however, was revealed only after ‘age over 60 years’ was identified and controlled for as a confounding variable. This result supports similar findings by other authors, who have shown statins to be neuroprotective [33] as well as important for stroke prevention [34]. 

Besides advanced age, one confounding factor that was not appropriately controlled for in neither the Katznelson *et al*. [22] nor the Redelmeier *et al*. [24] study was the frequency of atherosclerosis in their patient samples. The importance of controlling for atherosclerosis arises from two earlier studies that show that atherosclerosis is a significant, independent risk factor for delirium after cardiac surgery [35, 36]. The lower rate of delirium associated with the non-statin users in Katznelson *et al*. [22], for example, may also be attributed to a potentially lower rate of atherosclerosis in this population, and this could be the more major contributor to the pathophysiological state of delirium than the pharmacological actions of statins. Although atherosclerosis as a factor was not accounted for in Redelmeier *et al*. [24], a selection bias analysis was performed and showed that patients in their sample who were taking statins tended to be healthier than non-statin users. No description was given about the factors that were controlled for in this selection bias analysis; however, they concluded from this that hidden confounders would not explain the significantly independent, detrimental relationship that they found between statin use and delirium [24]. 

Other studies that included statins or lipid-lowering agents as variables in their databases failed to find a significant association with postoperative delirium use in either direction [9, 11]. This may be due in part to the much smaller sample sizes and lower frequency counts of patients who were taking statins in these studies (total *n* = 58 in Hudetz *et al*., [9]; total *n* = 113 in Burkhart *et al.*, [11]). 

Based on evidence from a systematic review, Kulik and Ruel [37] recommended that statins should be used for CABG patients and should ideally be started before surgery because of the beneficial cardiac and medical outcomes associated with perioperative statin use; but considering the current ambiguity regarding conclusions about the effects of statins on postoperative delirium, this suggestion should be carefully regarded. 

#### Antihypertensives

Antihypertensive drugs taken in the preoperative period, other than drugs with direct cholinergic receptor interactions, have not been linked to delirium after cardiac surgery in any study that was reviewed. Diuretics, calcium channel-blockers (CCBs), angiotensin-converting enzyme inhibitors (ACE-Is), β-blockers, angiotensin receptor blockers (ARBs) and nitrates have all failed to show a relationship with delirium in the studies published thus far [5, 7, 9, 10, 24]. It is unclear whether this is due to a true lack of association, or if these results are merely reflecting inappropriate study design or insufficient sample sizes.

### II Intraoperative Period

#### Diazepam

One class of drugs commonly used during surgery that has been implicated in the development of delirium after cardiac surgery is the benzodiazepines and their derivatives. Specifically, Santos *et al.* [5] showed that the use of 5-10 mg of oral diazepam versus 7.5-15mg midazolam for premedication 1 hour prior to anesthesia for nonemergency CABG procedures was associated with an increase in the incidence of delirium (*p* < 0.05)*.* However, when Santos *et al*. [5] performed stepwise logistic regression on all variables that achieved *p*-values ≤ 0.1 from univariate analysis, the use of pre-anesthetic diazepam was not identified to be an independently associated risk factor for delirium [5]. This therefore suggests that whatever association pre-anesthetic diazepam may have with delirium, it is likely mediated by another factor. 

#### Fentanyl

Fentanyl is frequently used to provide sedation and analgesia during cardiac surgical procedures because of its fast onset, short duration of action, and minimal cardiovascular depressive effects. A post-hoc analysis [11] performed on a randomized controlled trial [8] confirmed that patients who received higher doses of fentanyl during cardiac surgery under cardiopulmonary bypass were more likely to become delirious after the procedure than those who were administered lower doses of fentanyl during surgery. In this study fentanyl was the only intraoperative opioid that was used on patients during surgery [11]. For every 10μg/kg increase in fentanyl, an individual was approximately 3.4 times more likely to develop delirium (95% CI 1.41-8.14, *p* = 0.006) [11]. When these results were adjusted for the duration of surgery, the odds ratio increased to 4.9 times more likely (95% CI 1.72-13.8, *p* = 0.003) [11]. Importantly, this association was only significant when the doses of fentanyl were normalized and analyzed according to per kilogram of body weight [11]. The original randomized controlled trial by Gamberini *et al*. [8] had analyzed the amount of fentanyl received during surgery according to the median dose in milligrams per patient, without normalizing to body weight, and this failed to show a significant effect of fentanyl dose on delirium. Another study by Hudetz *et al*. [9] also did not evaluate dose of fentanyl per kilogram of body weight, and they too did not find a significant increase in the incidence of delirium with increasing doses. Apart from fentanyl, no other opioids (including sufentanil) have been reported in the literature on cardiac surgery to show a significant positive or negative association with postoperative delirium. 

The risk that fentanyl imparts is likely not due to its purported minor anticholinergic effects because recent *in vitro* studies have been unable to replicate earlier findings demonstrating that fentanyl displays serum anticholinergic activity [11, 31]. It is therefore unclear whether this association between fentanyl use and increased incidence of postoperative delirium described by Burkhart *et al.* [11] is mediated by the pharmacological action of fentanyl acting directly on the stereospecific opioid receptors in the CNS, if the respiratory depression caused by fentanyl is related to delirium, or if the downstream effect of sedation produced by fentanyl somehow affects a patient’s postoperative state of arousal, attention, and perception. None of the studies that included fentanyl in their investigations commented on the importance of adequate analgesia on the development of delirium, although the study by Gamberini *et *al. [8] reported that oral acetaminophen and intravenous morphine were administered in the postoperative period to keep pain at a minimum for study participants.

#### Ketamine 

The anesthetic agent ketamine is commonly regarded as a “dissociative” drug and has been frequently linked with emergence delirium [38]. However, it has unexpectedly been shown in one prospective randomized study by Hudetz *et al*. [9] to decrease the incidence of postoperative delirium after cardiac surgery. Patients were randomized to receive either a 0.5-mg/kg intravenous dose of ketamine or placebo, and all patients received fentanyl and etomidate for anesthetic induction. Patients who had received ketamine during anesthetic induction had an incidence of delirium that was approximately thirteen times lower than in patients who received placebo (*p* = 0.01) [9]. There was also an association between ketamine usage and the levels of C-reactive protein (CRP) in patients, whereby patients who were given ketamine also had lower CRP levels (*p* < 0.05) [9]. This led Hudetz *et al*. [9] to postulate that perhaps ketamine was conferring a neuroprotective effect by acting as an anti-inflammatory agent in the postoperative period to prevent cytokines from disrupting brain metabolism. 

Another possible reason for why ketamine may be associated with a decreased rate of postoperative delirium may be because using ketamine reduces opioid consumption in the postoperative period during the first 48-hours after cardiac surgery, as it is able to serve as an adequate adjunct to analgesic management for pain from sternotomies [39]. In Hudetz *et al*. [9], the amount of morphine consumed immediately after surgery and on postoperative day 1 did not significantly differ between the ketamine and placebo groups (*p* = 0.36 for the day of surgery, and *p* = 0.46 for postoperative day 1), nor did the proportion of patients who were given morphine in the postoperative period (*p* = 0.70). Mean total doses of fentanyl received during surgery were also compared, but did not statistically differ between the groups [9]. However, considering the results from Burkhart *et al.*’s [11] post-hoc analysis, it may have been crucial for Hudetz *et al*. [9] to calculate doses of fentanyl with respect to body weight instead of calculating the mean total amount of fentanyl consumed, since Burkhart *et al. *[11] were only able to find a significant association between fentanyl use and postoperative delirium when they normalized doses to body weight. Thus, the amounts of opioids that were used in each group may potentially be a confounding factor in this study.

### III Postoperative Period

#### Opioids

In a review of clinical risk factors for delirium by Sockalingam *et al*. [40], four studies identified postoperative morphine as a causative factor for postoperative delirium [41-44]. However, none of these studies focused exclusively on the cardiac surgery population, but instead were performed on general intensive care patients or non-cardiac surgery patients.

Within the cardiac surgery population, similar to preoperative use of opioids, postoperative use of opioids has not been shown to increase or decrease the rate of postoperative delirium. Specifically, two prospective studies found no statistically significant relationship between either average morphine-equivalent dose of opioids consumed over 3 postoperative days or opioid dose per kilogram of body weight and the incidence of delirium [11, 25]. Hudetz *et al*. [9] were also unable to find any relationship between the amount of morphine consumed on the day of surgery, the amount of morphine consumed on postoperative day 1, or the proportion of patients taking morphine postoperatively, with the incidence of delirium.

#### Sedatives

Choice of sedatives in the postoperative period may be critical for preventing postoperative delirium. The selective, centrally-acting α-2-adrenergic agonist, dexmedetomidine, has recently garnered interest due to its ability to provide adequate postoperative sedation and analgesia without producing excessive hypotension or the need for vasopressors, while reducing the risk of delirium after cardiac surgery [26]. Unlike the conventional sedatives propofol, midazolam, or morphine, dexmedetomidine is able to produce anxiolysis and sedation without producing significant respiratory depression [45], a property of particular desirability in cardiac surgery patients. It appears possible that this respiratory advantage of dexmedetomidine is directly related to its benefits in terms of delirium, as studies that have tested this novel sedative in cardiac surgery patients have suggested that dexmedetomidine’s effects on delirium are not simply due to its opioid-sparing properties [26]. A number of studies have reported that prolonged periods of intubation increase the risk of delirium by a factor of 1.10–7.90 times compared to shorter periods of intubation [46-48]. Thus, dexmedetomidine confers another potential advantage, in that patients may be extubated while remaining sedated, and that they may be maintained under sedation for as long as necessary [3] until homeostasis is recovered and pain and anxiety are kept under control [49]. 

A randomized controlled trial by Shehabi *et al*. [26] compared the incidence of delirium in patients receiving dexmedetomidine for postoperative sedation to patients receiving morphine. Interestingly, the authors found that the incidence of delirium was not significantly different between the two groups (8.6% delirious in dexmedetomidine group vs. 15.0% in morphine group, RR = 0.571, 95% CI 0.256-1.099, *p* = 0.088) [26]. Rather patients who were given dexmedetomidine for sedation, had shorter durations of delirium compared to their morphine-sedated counterparts, and they were extubated sooner (RR = 1.27, 95% CI 1.01-1.60, *p* = 0.040) [26]. Thus by this logic, it seems that choice of postoperative sedative may contribute to delirium by shaping its trajectory. 

In another randomized study by Maldonado *et al.* [3], postoperative sedation by dexmedetomidine (loading dose = 0.4μg/kg, infusion rate = 0.2-0.7μg/kg/hr) was compared to propofol (25-50μg/kg/min) and to midazolam (0.5-2mg/hr). Contrasting to Shehabi *et al*. [26], they found that the rate of delirium was significantly lower in patients receiving dexmedetomidine compared to propofol or to midazolam (3% delirium in dexmedetomidine group vs. 50% in propofol group and 50% in midazolam group; *p* < 0.001 for dexmedetomidine vs. propofol, and vs. midazolam) [3]. One problem in the studies by both Shehabi *et al.* [26] and Maldonado *et al*. [3] is that because it was not possible to include placebo groups for postoperative sedation, it is difficult to determine if the use of dexmedetomidine is actively reducing the rate of delirium in patients, or if in fact the use of propofol or midazolam is actively increasing it. 

One likely reason for the discrepancy between the rates of delirium associated with dexmedetomidine use reported by Shehabi *et al*. [26] and Maldonado *et al*. [3] is due to the differences in methods for diagnosing delirium. Shehabi *et al*. [26] used a standardized diagnostic algorithm, the CAM-ICU, while Maldonado *et al*. [3] used formal clinical diagnoses based on DSM-IV-TR criteria. As the CAM-ICU has lower sensitivity and specificity compared to formal diagnoses of delirium by clinicians [50], the rates reported by Shehabi *et al*. [26] may be underestimated compared to Maldonado *et al*. [3]. 

#### Inotropes

While inotropes are commonly used in the postoperative period to maintain hemodynamic stability in cardiac surgery patients, only one study that met the inclusion criteria looked at their effects on delirium. Norkiene *et al*. [51] reported that when inotropes are used in the postoperative period for more than 12 hours, patients were approximately 8-times more likely to become delirious (OR = 8.04, 95% CI 1.1-60.6; *p* = 0.002). This was found to be one of the strongest independent predictors of delirium in this sample after using multivariate regression analysis. No other study in the context of cardiac surgery has yet reproduced this finding. 

It is especially important, when considering the influence of inotropes on delirium, to consider if this association may be confounded by indication. The reason for this is because problems with cardiac contractility can affect cerebral perfusion, and perfusion abnormalities have been observed by Single Photon Emission Computed Tomography (SPECT) in the brains of older hospitalized delirious patients [52]. With respect to the Norkiene *et al*. [51] study, the mean New York Heart Association functional classification score (NYHA), the proportions of individuals with previous myocardial infarctions, and the proportions of individuals with intra-aortic balloon pumps (IABPs) before surgery did not significantly differ between delirious and non-delirious groups (*p* = 0.08, *p* = 0.05, *p* = 0.07 respectively). However, delirious and non-delirious individuals did have significantly different ejection fractions prior to surgery, with delirious individuals having significantly lower mean ejection fractions in the preoperative period (*p* = 0.005) [51]. Thus, if the authors did not control for this factor in multivariate analysis, differences in preoperative ejection fraction may be one potential confounding factor in this study, among others.

### IV Agents for Prophylaxis

#### Risperidone

As previously mentioned, preoperative use of antipsychotics was shown by Redelmeier *et al*. [24] to increase a patient’s risk of developing delirium after surgery. However, antipsychotic drugs are also often used to alleviate the psychotic symptoms of delirium, including perceptual changes, agitation and paranoid ideation, following many different kinds of surgery [49]. The most commonly used antipsychotic for treating delirium off-label is haloperidol, although loxapine, quetiapine, olanzapine, methotrimeprazine, and risperidone have also been used. There is very little definitive evidence for the use of antipsychotic drugs in postoperative cardiac surgical patients. Risperidone, an antagonist of dopamine D_2_ and serotonin 5-HT_2_ receptors, is the only agent that has been studied in the context of delirium after cardiac surgery for its role as a potential prophylactic agent. The reason this drug was selected for investigation is presumably because of its relatively long half-life, which can be up to 20-hours long in poor metabolizers, and this is due to the similar pharmacology of its active metabolite, 9- hydroxyrisperidone [54]. In its use as a preventative agent for delirium, it has also been reported to produce relatively few adverse effects [6]. In the latter study, when risperidone was administered at a single dose of 1mg sublingually immediately following awakening from sedation in the intensive care unit after cardiac surgery, it lowered the incidence of delirium to one-third the incidence that was seen in patients receiving placebo [6]. Unlike dexmedetomidine, which may be mediating its beneficial effects in delirium by producing sedation without respiratory depression in elderly patients, the relatively low risperidone doses used in the prevention of delirium make excess sedation less of a risk.

Despite these findings, the future use of risperidone for preventing delirium in cardiac surgery patients remains uncertain. Adverse effects of risperidone, such as hypotension, symptomatic orthostasis, and cardiac arrest, have been reported to be associated in particular with cardiovascular disease and its treatment [55], although these side-effects were more common in the 1990s when doses of the drug used for the treatment of psychosis were substantially higher than are typically used nowadays. Compared to intravenous administration of ketamine and dexmedetomidine during anesthesia, another challenge to using risperidone for prophylaxis immediately upon awakening is maintaining adequate absorption of the drug in its oral form. A significant proportion of patients experience nausea and emesis after cardiac surgery, interfering with effective absorption of oral prophylactic treatments. Additionally, while it is possible to administer risperidone sublingually, the site of absorption is unknown (i.e., sublingual, versus buccal, versus gastrointestinal) but since it peaks 1-2 hours after oral administration, oral absorption is still required. 

While it may seem contradictory for antipsychotics to be both beneficial and harmful for delirium (as in the case in Redelmeier *et al*. [24]), one explanation for this is that the preoperative, detrimental association that was observed by Redelmeier *et al*. [24] was likely confounded by indication since this was not controlled for in their regression model. Rather than attribute this discrepancy to the specific type of antipsychotic medication or to dose, the suggestion that this association is confounded by indication is supported by the fact that cognitive impairment and certain mental illnesses like depression have been repeatedly shown to be associated with delirium after cardiac surgery [7, 14, 22]. 

#### Rivastigmine

Along with studies showing the potentially beneficial effects of taking preoperative statins, intraoperative ketamine, and postoperative dexmedetomidine, the cholinesterase inhibitor rivastigmine, has also been tested for its efficacy to prevent delirium after cardiac surgery. The rationale for this study was based on the “cholinergic deficiency” theory of delirium. This theory, which postulates that delirium is caused by insufficient levels of acetylcholine in the brain, is derived from the observations by Tune *et al*. [56] that showed that increased serum levels of anticholinergic drugs were associated with an increased occurrence of postoperative delirium. Given this finding, cholinesterase inhibitors, which prevent the breakdown of acetylcholine within the neural synapse, should have the potential to prevent delirium. However, the only cholinesterase inhibitor to be investigated in the cardiac surgery setting in recent years is rivastigmine.

In a randomized, double-blind, prospective interventional study, short-term oral rivastigmine was administered to CPB patients the evening before their procedures, and was continued every 6 hours following surgery for 6 days [8]. Interestingly, this regimen failed to lower the incidence of delirium, with the incidence in rivastigmine-treated patients remaining at 32% compared to 30% in placebo-treated patients [8]. As the authors discussed, it is likely that in this case, the dose of rivastigmine that was used (1.5mg, three times a day, every 6 hours) was a limiting factor in demonstrating a significant advantage. They suggested that since side effects occurred in approximately equal rates between the rivastigmine and placebo groups, it is likely that the dose of rivastigmine that was used was too low [8].

Another, albeit less-detailed investigation of cholinesterase inhibitors was performed by Redelmeier *et al*. [24], who included this variable in their retrospective cohort study of risk factors for postoperative delirium. Redelmeier *et al.* [24] found that rather than decreasing the risk for delirium as Tune *et al*. [56] and Gamberini *et al*. [8] had rationalized, patients in their patient sample who were taking this class of drugs preoperatively were in fact at increased risk for developing delirium after surgery. These results from the Redelmeier *et al*. [24] study were obtained from univariate analysis, so they were entered into a multivariate model to control for age, sex, duration of surgery, individual medications, and surgery type, and still the results remained significant (adjusted odds ratio, 1.28; 95% CI, 1.12-1.46). One important factor that Redelmeier *et al*. [24] failed to include in the regression model is indication – cholinesterase inhibitors are routinely prescribed for cognitive decline, and these types of conditions have been shown in many instances to be associated with postoperative delirium [7, 14, 22]. However, because the Redelmeier *et al. *[24] study was a retrospective cohort analysis and used a different diagnostic technique than Gamberini *et al*. [8], it may be inappropriate to compare these findings. Thus, the effectiveness of cholinesterase inhibitors in preventing delirium is still inconclusive and requires further investigation.

## CONCLUSION 

Two single drugs (intraoperative fentanyl and ketamine), and two classes of drugs (preoperative antipsychotics and postoperative inotropes) have thus far been identified to be independently associated with delirium after cardiac surgery. Specifically, preoperative antipsychotics, intraoperative fentanyl and postoperative inotropes are associated with higher rates of delirium, while intraoperative ketamine is associated with a lower rate of delirium. Additionally, the administration of one drug (risperidone) has been shown to be effective for preventing delirium after cardiac surgery. Another seven classes of drugs (preoperative antihypertensives, anticholinergics, antidepressants, benzodiazepines, opioids, and statins, and postoperative opioids) and three single drugs (intraoperative diazepam, and postoperative dexmedetomidine and rivastigmine) have mixed findings in relation to delirium after cardiac surgery.

From the results of this review, it is clear that drugs taken perioperatively by cardiac surgery patients have either direct or indirect influence on the outcome of delirium. However, given the present state of delirium research, relatively little is known about how the large number of drugs administered to cardiac surgery patients before, during and after surgery contributes to postoperative delirium. Most of the studies that have recorded perioperative drug use are very broad in their approach and do not include analysis on specific drugs. In addition, the literature on delirium after cardiac surgery is heterogeneous and differs quite substantially in terms of designs, methodologies, definitions and diagnostic instruments, making it difficult to compare the results. For instance, investigators’ selection of covariates for their regression model could significantly change the outcomes and the conclusions that could be made. As for whether or not specific covariates should be consistently accounted for in future studies, investigators should be attentive to control for the factors of age, sex, and any clear confounders of indication; however, it is always important to select covariates based on results of current research. None of the findings on the drugs that have been reported to be associated with delirium, including the potentially prophylactic drugs, have been reproduced in the cardiac surgical population. For this reason, any general conclusions about the relationship of these drugs to postoperative delirium should be considered with these limitations in mind. Future studies on the relationship between perioperative drug use and postoperative delirium clearly are necessary; in particular, these should consider statistical solutions for taking into account factors that influence drug action within the individual, such as drug dose and efficiency of drug metabolism, as some studies that were reviewed obtained significant results only when drug doses were normalized to body weight. Although the evidence for the role of drugs in the etiology of postoperative delirium may not yet be completely conclusive, the data available to date indicate that this should be considered an important aspect of the management of cardiac surgery patients at risk for delirium throughout the course of care.

## Figures and Tables

**Table 1.** Summary of the Studies that have Recorded Perioperative Drug use in Relation to Delirium after Cardiac Surgery

Reference	Sample Description	Surgeries	Assessment and Rate of Delirium	Drugs Studied	P-Values^a^^b^		Odds	
**Prospective Observational Studies**								
Santos *et al*., [5]	Average age = 71 yrs *n *= 220	Non-emergency CABG on CPB	DSM-IV, 33.6%	Collected data on preoperative use of diuretics, CCBs, b-blockers, ACEIs, nitrates, PPIs; anesthetic premedication with either diazepam or midazolam	Diuretics CCBs b-blockers ACEIs Nitrates PPIs **Diazepam**	0.080 0.518 0.179 0.088 0.632 0.865 **<0.05**	NA	
**Objective:** To determine risk factors for delirium after CABG								
Rudolph *et al*., [7]	Average age = 74 yrs *n* = 42	CABG, valve replacement, combined CABG-valve surgery on CPB	MMSE, DSI, MDAS, CAM, 29%	Collected data on preoperative use of aspirin, NSAIDs, steroids, b-blockers, ACEIs, ARBs, CCBs, nitrates, diuretics	NA (but reported no significant differences in preoperative medication use between patients with and without delirium)		NA	
**Objective:** To compare changes in the levels of inflammatory markers in patients with and without delirium after cardiac surgery								
Afonso *et al*., [10]	Average age = 66 yrs *n =* 112	Cardiac or thoracic aortic surgeries on CPB	RASS, CAM-ICU, 34%	Collected data on preoperative use of nitrates^c^, benzodiazepines, SSRIs and ACEIs	ACEI	0.91	NA	
**Objective:** To produce a predictive model for delirium after cardiac surgery								
Katznelson *et al*., [22]	Average age = NA (36% were < 60 yrs old, and 64% were ³ 60 yrs old) *n *= 1,059	CABG, valve replacements, combined CABG-valve replacement surgery on CPB	CAM-ICU, 11.5%	Looked at preoperative use of statins and perioperative benzodiazepine and opioid use	**Statin**	**<0.01**	**Statin**	Odds Ratio^d^ (95% CI) **0.54 (0.35-0.84)**
**Objective:** To demonstrate an association between preoperative statin use and delirium after cardiac surgery								
Tan *et al*., [25]	Average age = 63 yrs *n* = 53	CABG, valve replacements, combined CABG-valve surgery on CPB	CAM, MDAS, MMSE, 23%	Collected data on preoperative ‘chemical dependency’; and use of ‘other’; anticholinergic medications (neither variable was defined); also recorded postoperative morphine equivalents over POD 1-3	Chemical dependency ‘Other’; anti-cholinergic agents Morphine equivalents, POD 1-3	0.18 0.10 0.76	‘Other’; anticholinergic agents Morphine equivalents, POD 1-3	Risk Ratio (95% CI) 2.31 (0.85-6.31) 1.00 (0.99-1.01)
**Objective:** To determine factors associated with and rate of delirium after cardiac surgery								
**Prospective Observational Studies**								
Tully *et al*., [27]	Average age = 65 yrs *n* = 158	Elective CABG, combined CABG-valve surgery on CPB	DSI, DSM-IV-TR, 31%	Collected data on preoperative use of anticholinergic drugs, SSRIs, tricyclic antidepressants	Anticholinergic drugs **SSRI** Tricyclic antidepressant **Composite of drugs (anticholinergics, SSRIs, and/or tricyclics)**	0.31 **0.01** 0.65 **0.01**	**Composite of drugs **	Odds Ratio (95% CI) **5.12 (1.46-17.94)**
**Objective:** To determine an association between preoperative affective disorders or Type D personality with delirium after cardiac surgery								
Koster *et al*., [29]	Average age = 70 yrs *n *= 112	Elective cardiac surgery with or without CPB	DOS, DSM-IV, 21%	Collected data on preoperative opioid use	Opioids	1.00	NA	
**Objective:** To determine the predictive validity of a risk checklist for delirium after cardiac surgery								
Norkiene *et al*., [51]	Average age = 71 yrs *n *= 1,367	CABG on CPB	DSM-IV, 3.1%	Collected data on postoperative use of inotropes for more than 12 hours	**Inotropes > 12hrs**	**0.002**	**Inotropes > 12hrs**	Odds Ratio (95% CI) **8.04 (1.1-60.6)**
**Objective:** To identify the incidence and risk factors for delirium after cardiac surgery								
**Retrospective Chart Review**								
Redelmeier *et al*., [24]	Average age = 74 yrs *n* = 284,158	Elective cardiac, thoracic, neurosurgical, vascular, musculoskeletal, abdominal, retroperitoneal, lower urogenital, breast and skin, external head and neck, ophthalmologic, and unclassified surgeries	ICD, 1.1%	Outpatient use of two or more of: atorvastatin, simvastatin, pravastatin, lovastatin, fluvastatin, rosuvastatin, and/or cerivastatin, with at least one having been prescribed within 90-days before surgery; also preoperative neuropsychiatric agents, nonstatin lipid-lowering agents, antihypertensives, loop diuretics, cardiovascular agents, anticoagulants, vascular agents, other common drugs	**Statins** **Cholinesterase inhibitor** **Antipsychotic** **Antidepressant** **Benzodiazepine** ACEI ARB Thiazide diuretic CCB Furosemide Digoxin Spironolactone Nonstatin lipid-lowering agent Anticoagulant Antiplatelet Pentoxifylline Hypoglycemics Insulin Bronchodilator Allopurinol Levothyroxine Glucocorticoid Gastric acid suppressant Anti-osteoporosis Glaucoma eye drops	**< 0.001** **< 0.001** **< 0.001** **< 0.001** **< 0.001** 0.43 0.77 0.29 0.21 0.71 0.60 0.59 0.21 0.16 0.95 0.44 0.57 0.59 0.34 0.10 0.99 0.56 0.80 0.89 0.39	**Statins** **Cholinesterase inhibitor** **Antipsychotic** **Antidepressant** **Benzodiazepine** ACEI ARB Thiazide diuretic CCB Furosemide Digoxin Spironolactone Nonstatin lipid-lowering agent Anticoagulant Antiplatelet Pentoxifylline Hypoglycemic Insulin Bronchodilator Allopurinol Levothyroxine Glucocorticoid Gastric acid suppressant Anti-osteoporosis Glaucoma eye drops	Odds Ratio^e^ (95% CI) **1.28 (1.12-1.46)^f^** **3.99 (2.26-7.05)** **1.57 (1.26-1.95)** **2.01 (1.75-2.25)** **1.40 (1.28-1.53)** 0.96 (0.87-1.06) 1.05 (0.73-1.52) 0.92 (0.79-1.07) 0.94 (0.85-1.04) 1.03 (0.89-1.18) 0.96 (0.83-1.11) 1.09 (0.80-1.47) 0.80 (0.56-1.14) 1.14 (0.95-1.38) 1.01 (0.68-1.52) 1.16 (0.80-1.69) 0.96 (0.83-1.11) 0.93 (0.73-1.20) 1.06 (0.94-1.20) 0.83 (0.67-1.03) 1.00 (0.88-1.14) 0.94 (0.77-1.15) 0.99 (0.90-1.08) 1.01 (0.86-1.18) 0.94 (0.80-1.09)
**Objective:** To determine if the use of statins was associated with a higher rate of delirium after cardiac and non-cardiac surgeries								
**Post-Hoc Analysis of a Randomized Controlled Trial**								
Burkhart *et al*., [11]	Average age = 74 yrs *n* = 113	Elective cardiac surgery with CPB	CAM, 30%	Collected data on preoperative use of statins; intraoperative amount of fentanyl; postoperative use of metoclopramide and tropisteron, and postoperative amount of opioids received per kilogram of body weight	Statins **Fentanyl dose, per 10-µg/kg increase** Opioid dose, per 1-mg/kg increase	0.8 **0.006** 0.6	Statins **Fentanyl dose, per 10-µ>g/kg increase** Opioid dose, per 1-mg/kg increase	Odds Ratio (95% CI) 1.1 (0.49-2.48) **3.4 (1.41-8.14)** 1.5 (0.29-8.18)
**Objective:** To identify modifiable risk factors for delirium after cardiac surgery								
**Randomized Controlled Trial (Open-Label, Treatment-Controlled)**								
Maldonado* et al*., [3]	Average age = 58 yrs *n* = 90	Elective valve procedures on CPB	DSM-IV-TR, Overall rate, 34% Dexmedeto-midine cohort, 3% Propofol cohort, 50% Midazolam cohort, 50%	Compared rate of delirium with postoperative sedation by dexmedetomidine to sedation by propofol, or midazolam (in each sedation protocol, also looked at differences in intraoperative amount of fentanyl and midazolam, postoperative amount of fentanyl and total morphine equivalents, and postoperative use of antiemetics, lorazepam, and haloperidol)	**Dexmedetomidine/propofol/midazolam** **Midazolam/dexmedetomidine** **Propofol/dexmedetomidine**	**< 0.001** **<0.001** **<0.001**	**Midazolam (vs. dexmedetomidine)** **Propofol (vs.dexmedetomidine)**	Odds Ratio^g^ (95% CI) **28.6 (4.7-262.5)** **29.6 (4.8-280.6)**
**Objective:** To determine the effects of postoperative sedation on delirium after cardiac surgery								
**Randomized Controlled Trial (Double-Blind, Treatment-Controlled)**								
Shehabi *et al*., [26]	Average age = 71 yrs *n* = 299	CABG, valve replace- ments, combined CABG-valve surgery on pump	CAM-ICU, Overall rate, 11.7% Dexmedetomidine cohort, 8.6% Morphine cohort, 15%	Compared rate of delirium with postoperative sedation by dexmedetomidine to sedation by morphine (used open-label morphine in dexmedetomidine group to titrate for analgesia and open-label propofol in morphine group to titrate for sedation)	Dexmedetomidine/ morphine	0.088	Dexmedetomidine (vs. morphine)	Risk Ratio (95% CI) 0.57 (0.26-1.1)^h^
**Objective:** To compare the incidence of delirium in patients postoperatively sedated with dexmedeto- midine or morphine after cardiac surgery								
**Randomized Controlled Trials (Double-Blind, Placebo-Controlled)**								
Prakanrattana and Prapaitrakool, [6]	Average age = 61 yrs *n* = 126	Elective cardiac surgery on CPB	CAM-ICU, Risperidone cohort, 11.1% Placebo cohort, 31.7%	Investigated the effectiveness of postoperative risperidone prophylaxis (one dose of 1 mg risperidone or placebo immediately upon awakening from sedation in the ICU)	**Risperidone/ placebo**	**0.009**	**Risperidone (vs. placebo)**	Risk Ratio (95% CI) **0.35 (0.16-0.77)**
**Objective:** To evaluate the efficacy of immediate postoperative risperidone for prevention of delirium after cardiac surgery								
Gamberini *et al*., [8]	Average age = 74 yrs *n* = 113	CABG, valve replace- ments, with or without CPB	CAM, Rivastigmine cohort, 32% Placebo cohort, 30%	Investigated the effectiveness of a rivastigmine prophylaxis regimen (3 doses of 1.5 mg o.d. rivastigmine or placebo starting on evening before surgery until POD 6)	Rivastigmine/ placebo	0.8	Rivastigmine (vs. placebo)	Risk Ratio (95% CI) 1.08 (0.62 -1.90)
**Objective:** To evaluate the efficacy of rivastigmine prophylaxis regimen for the prevention of delirium after cardiac surgery								
Hudetz *et al*., [9]	Average age = 64 yrs *n* = 58	Elective CABG, valve replacements, valve repairs on CPB	Intensive Care Delirium Screening Checklist (based on DSM-IV), Ketamine cohort, 3% Placebo cohort, 31%	Investigated the effectiveness of intraoperative ketamine for prophylaxis (0.5 mg/kg i.v. ketamine or 0.9% saline placebo during anesthetic induction along with fentanyl and etomidate)	**Ketamine/placebo**	**0.01**	**Placebo (vs. ketamine)**	Odds Ratio (95% CI) **12.6 (1.5-107.5)**
**Objective:** To evaluate the efficacy of intraoperative ketamine for the prevention of delirium after cardiac surgery								

*CABG*, coronary artery bypass graft surgery; *CPB*, cardiopulmonary bypass; *DSM-IV-(TR)*, diagnostic and statistical manual of mental disorders fourth, (text-revised) edition; *CCB*, calcium channel blocker; *ACEI* angiotensin-converting enzyme inhibitor; *PPI*, proton-pump inhibitor; *MMSE*, mini-mental state examination; *DSI*, delirium symptom interview; *MDAS*, memorial delirium assessment scale; *CAM-(ICU)*, confusion assessment method (intensive care version); *NSAID*, non-steroidal anti-inflammatory drug; *ARB*, angiotensin receptor II blocker; *RASS*, Richmond agitation-sedation scale; *SSRI*, selective serotonin reuptake inhibitor; *CI*, confidence interval; *POD* postoperative day; *DOS*, delirium observation screening scale; *ICD*, international classification of diseases; *ICU*, intensive care unit; *o.d., omne in die; i.v*., intravenously; *NA,* not available

a With respect to delirium after surgery

b Calculated based on the proportions of patients on these drugs, unless otherwise specified

c Did not analyze preoperative nitrates, benzodiazepines or SSRIs because only one patient was taking each of these drugs

d Adjusted for age, preoperative depression, preoperative renal dysfunction, complex cardiac surgery, perioperative intra-aortic balloon pump, and massive blood transfusion

e Adjusted for age, sex, duration of surgery, individual medications, and type of surgery

f Adjusted for age, sex, social status, prior admissions, duration of surgery, individual medications, type of surgery

g Adjusted for other sedatives, age, ASA score (American Society of Anesthesiologists Physical Status Classification System score), male sex

h While Shehabi *et al*. [26] failed to find a statistically significant difference in the incidence of delirium between morphine- and dexmedetomidine-treated patients, they did find that compared to delirious morphine-treated patients, dexmedetomidine patients who did become delirious had shorter durations of delirium (*p* = 0.0317), were extubated earlier (*p* = 0.04), had fewer episodes of systolic hypotension (*p*=0.006), required less norepinephrine (*p* = <0.001), but had more bradycardia (*p* = 0.006).

**Table 2.** Summary of Drugs that have been Shown to be Independently Associated with Delirium after Cardiac Surgery (Drug Names are Given as they were Reported in the Original Studies)

Drug	Effect on Rate of Delirium^a^	References
**Preoperative Period**		
Statins	Mixed findings	[11, 22, 24]
Non-statin lipid lowering agents	No effect	[9, 24]
Cholinesterase inhibitors	No effect	[8, 24]
Anticholinergic agents	Mixed findings	[25, 27]
Antipsychotics	Increase	[24]
Antidepressants	Mixed findings	[24, 27]
SSRI	Mixed findings	[10, 27]
Benzodiazepines	Mixed findings	[10, 24]
Opioids	No effect	[29]
Diuretics	No effect	[5, 7, 9, 24]
CCBs	No effect	[5, 7, 9, 24]
β-blockers	No effect	[5, 7, 9, 24]
ACEIs	No effect	[5, 7, 9, 10, 24]
ARBs	No effect	[7, 24]
Nitrates	No effect	[5, 7, 9, 10, 24]
PPIs	No effect	[5, 24]
Digoxin	No effect	[24]
Anticoagulants	No effect	[24]
Antiplatelet agents	No effect	[24]
Pentoxifylline	No effect	[24]
Oral hypoglycemic agents	No effect	[24]
Insulin	No effect	[24]
Bronchodilator	No effect	[24]
Allopurinol	No effect	[24]
Levothyroxine	No effect	[24]
Steroids	No effect	[7, 24]
Anti-osteoporosis agents	No effect	[24]
Glaucoma eye drops	No effect	[24]
Aspirin	No effect	[7]
NSAIDs	No effect	[7]
**Intraoperative Period**		
Diazepam	No effect^b^	[5]
Ketamine	Decrease	[9]
Fentanyl, dose/patient	Mixed findings	[8, 9]
Fentanyl, dose/kg body weight	Increase	[11]
**Postoperative Period**		
Dexmedetomidine	Mixed findings	[3, 26]
Morphine, dose/patient	No effect	[9, 25, 26]
Opioids, dose/kg body weight	No effect	[11]
Inotropes > 12 hours	Increase	[51]
**Prophylactic Regimens**		
Risperidone	Decrease	[6]
Rivastigmine	No effect	[8]

*SSRI*, selective serotonin-reuptake inhibitor; *CCB*, calcium channel blocker; *ACEI*, angiotensin-converting enzyme inhibitor; *PPI*, proton-pump inhibitor; *ARB*, angiotensin receptor blocker; *NSAID*, non-steroidal anti-inflammatory drug

a Effect is based on proportions of delirious and non-delirious patients taking the drug, unless otherwise stated (e.g., the effect of postoperative opioids on delirium was analyzed based on dose/kg body weight)

b Diazepam was associated with an increased rate of delirium in univariate analysis, but lost this association with stepwise logistic regression

## Footnotes

1 While heart transplants were included for review, only the study by Afonso *et al*. [10] included patients who had received heart transplants (*n* = 1). For this reason, the effect of heart transplant on delirium is not given considerable weight in this review.
